# Chiari Pelvic Osteotomy and Femoral Varus Osteotomy for Recurrent Hip Dislocation in a Patient With Complete Lower Extremity Paralysis Due to Spina Bifida at the Thoracic Level

**DOI:** 10.7759/cureus.72275

**Published:** 2024-10-24

**Authors:** Yasuhiro Yamanaka

**Affiliations:** 1 Orthopedics, Sapporo City General Hospital, Sapporo, JPN

**Keywords:** chiari pelvic osteotomy, femoral varus osteotomy, lower extremity paralysis, recurrent hip dislocation, spina bifida, thoracic level, wheelchair basketball

## Abstract

Spina bifida can result in lower-extremity motor and sensory deficits, often leading to hip joint dislocation, a significant disability. The optimal surgical approach for hip dislocation in spina bifida patients remains a subject of debate. A 12-year-old girl with thoracic-level spina bifida experienced recurrent hip dislocations, significantly impacting her daily life and sports activities. At the age of 13, she underwent a femoral varus osteotomy. However, after six months, her hip dislocated again. At the age of 14, she underwent a Chiari pelvic osteotomy in addition. Six years later, she exhibited no limitations in her daily activities and sports participation. We report the first successful treatment of recurrent hip dislocation in a spina bifida patient with thoracic-level involvement, utilizing a combination of femoral varus osteotomy and pelvic osteotomy. Prior to surgical intervention, not only a comprehensive assessment of the patient's physical examination but also an assessment of their goals and living environment are essential.

## Introduction

Spina bifida is a congenital condition that affects the development of the spinal cord and can lead to a range of neurological problems. Depending on the type and severity of spina bifida, patients may experience a variety of symptoms, including paralysis or weakness in the legs, loss of bladder or bowel control, abnormal curvature of the spine, hydrocephalus, and developmental delays [1,2]. The purpose of orthopedic treatment in spina bifida is to support the patient's independent activities of daily living and to improve their quality of life [3]. Dislocation of the hip joint is a common problem for children with spina bifida, and it can be caused by a variety of factors, including muscle weakness, femur deformity, and deficiencies in the acetabulum [4]. Surgical intervention may be necessary for spina bifida patients with a dislocated hip joint if other conservative treatments have failed. The choice of surgical procedure will depend on the specific needs of the patient, the degree of hip instability, and the surgeon's experience and expertise. It's important to note that surgical intervention for spina bifida patients with a dislocated hip joint carries some risks, including infection, bleeding, nerve damage, and re-dislocation. Patients should discuss the risks and benefits of surgery with their surgeon and make an informed decision based on their individual circumstances. However, previous information on the outcomes of this surgery in the literature is quite limited, and no universal standards, guidelines, or reviews were found. We present a case of recurrent dislocation of the hip treated with femoral varus osteotomy and Chiari pelvic osteotomy to enhance the stability of the joint. At the time of the final follow-up, the patient had a good clinical result.

## Case presentation

**Figure 1:**
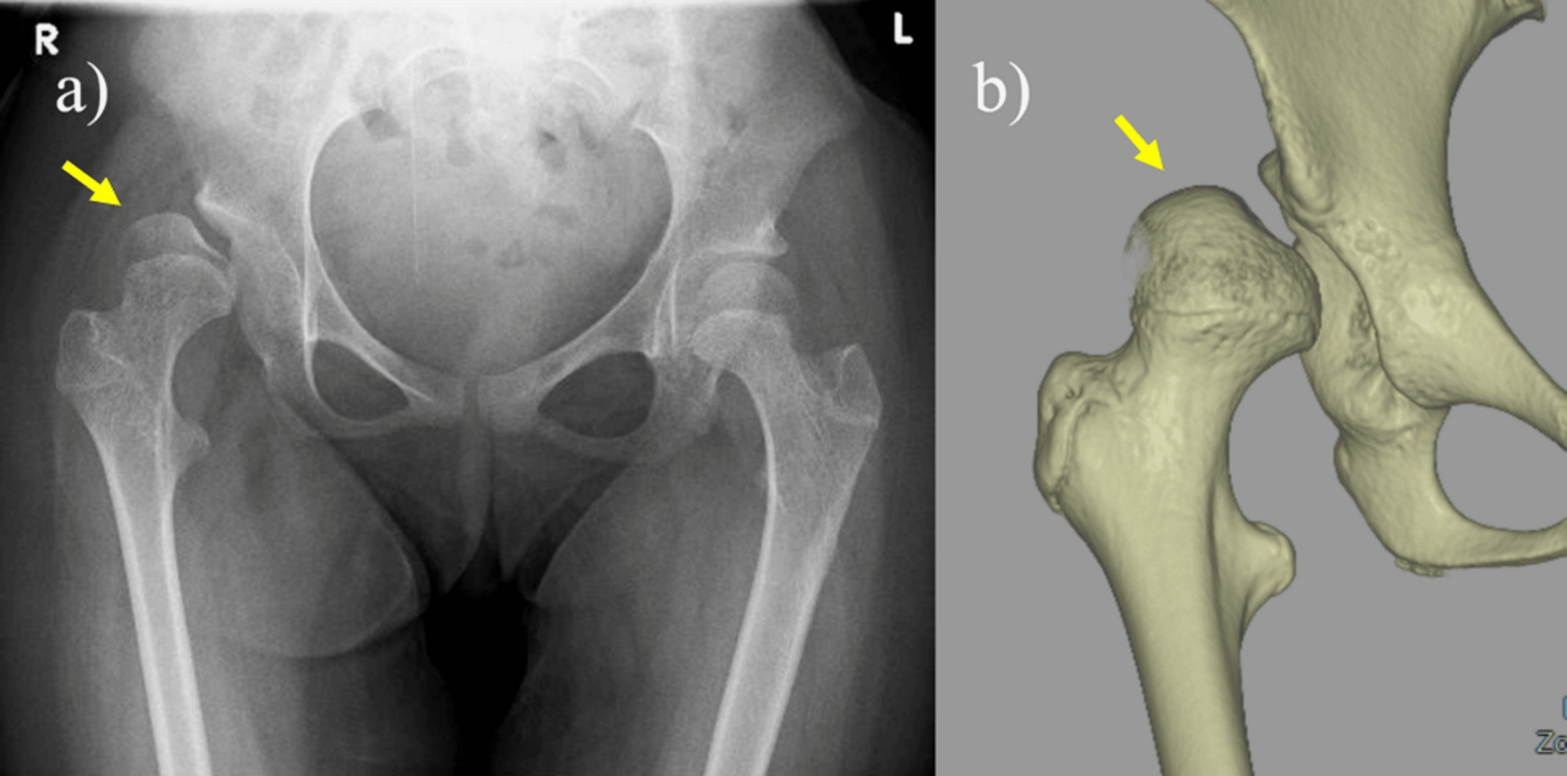
a) Anteroposterior (AP) radiograph and b) a computerized tomography (CT) scan shows a deficient acetabulum and dislocation of the right hip.

A 12-year-old girl with thoracic-level spina bifida was referred to our orthopedic department for treatment of recurrent dislocation of her right hip. She experienced discomfort when her hip joint was dislocated and had difficulty changing clothes by herself, especially putting on pants, due to hip instability. Physical examination revealed a girl with a body mass index of 27.4kg/m2, a weight of 40 kg, and a height of 120 cm. All higher mental functions and cranial nerves were normal. Upon neurological examination, the patient had a diminished sensation of pinprick and a light touch below her umbilicus, and there was complete flaccid paralysis of both lower extremities. She had a trunk dysfunction and was unable to sit up on her own. Deep tendon reflexes revealed negative for both knee jerk and Achilles tendon reflexes bilaterally. Sensory impairment was noted below the T8-10 level with urinary retention. She was unable to walk and used a wheelchair in her daily life. Her medical history was relevant for the removal of a spinal lipoma soon after birth, a VP shunt at 7 days, and spinal fusion surgery for severe kyphosis and scoliosis at the ages of 6 and 8 years. Antero-posterior (AP) radiograph and computerized tomography (CT) scan showed a deficient acetabulum and dislocation of the right hip (Figure 1).　We discussed her physical condition, wills, and living environment. The discussion was attended by the attending physician, pediatric orthopedic surgeon, parents, and orthopedic professor from the affiliated medical university because surgical indication for paralytic hip dislocation was controversial.

**Figure 2:**
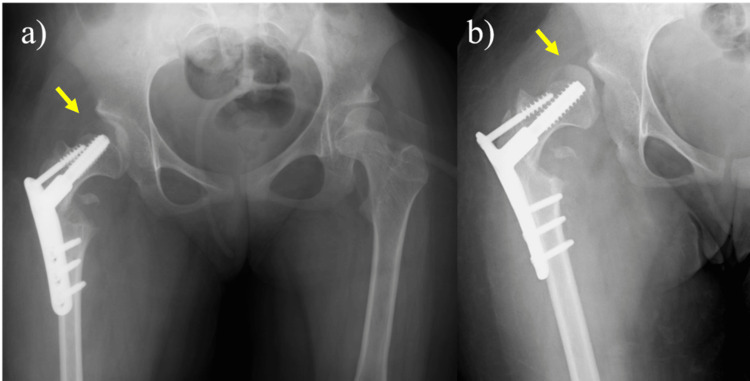
a) At the age of 13, a femoral varus osteotomy was performed, b) the right hip dislocated after 6 months.

**Figure 3:**
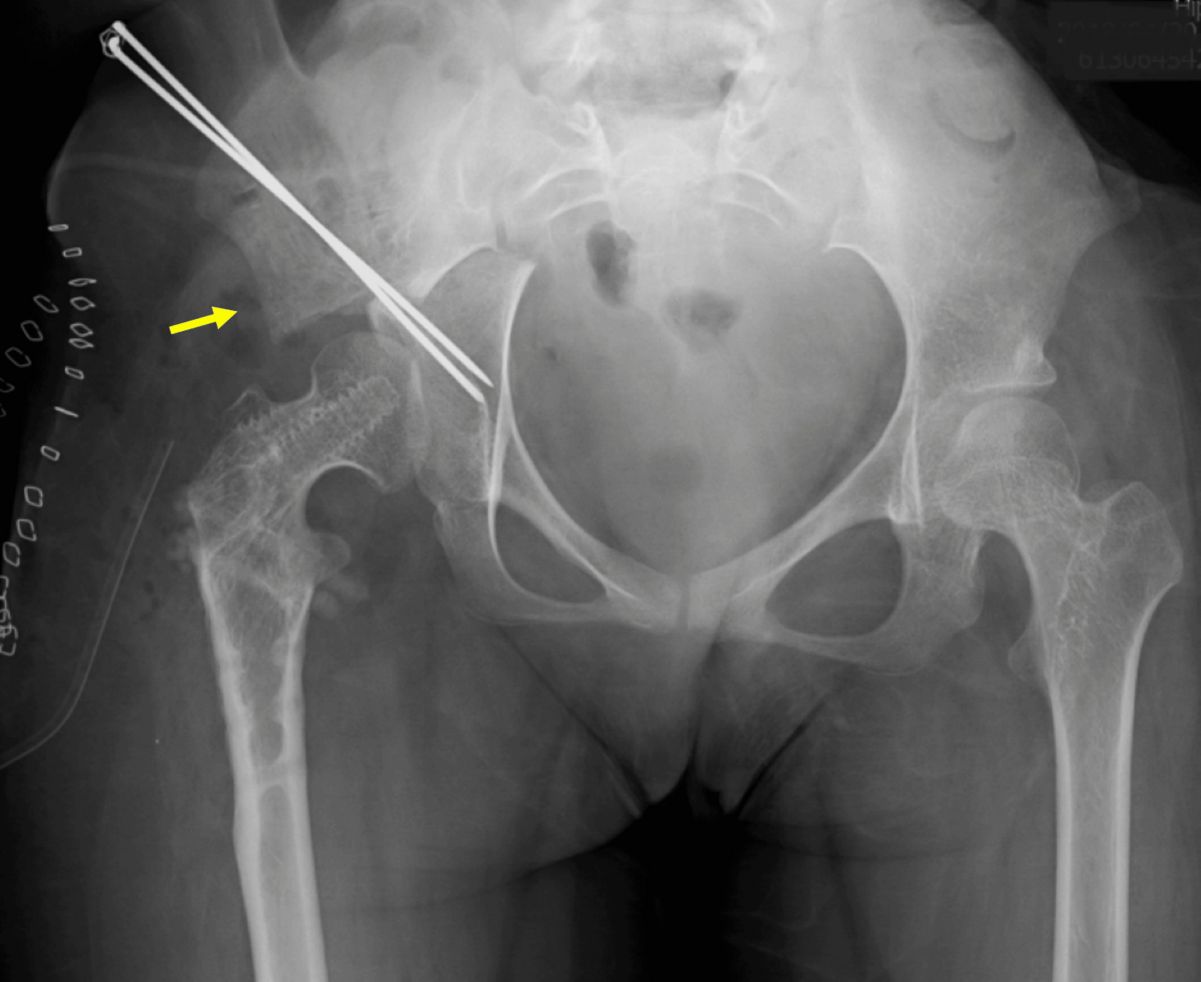
Chiari pelvic osteotomy was performed at the age of 14

Finally, we decided to perform a femoral varus osteotomy [5], because it can be performed with a relatively low-invasive surgery, to improve her hip stability. At the age of 13, she underwent surgery on her hip joint. A longitudinal skin incision was made over the greater trochanter, and after dissection of the deep fascia, the greater and lesser trochanters were exposed posteriorly. The external rotators and quadratus muscle were retained, and the medial femoral circumflex artery was preserved. A reciprocating saw was used with a crescentic osteotomy guide for the correction, which was performed under fluoroscopy. The femoral head was stabilized using a compression hip screw. A hip spica cast was applied for four weeks postoperatively. The postoperative course was uneventful for six months; however, her right hip dislocated again (Figure 2). We concluded that the femoral osteotomy was insufficient to maintain hip stability. At the age of 14, her hip joint was operated again. Chiari pelvic osteotomy was performed through an Ollier lateral U approach along with a trochanteric osteotomy [6]. The greater trochanter with its tendinous insertion was then retracted proximally to expose the entire joint capsule. The dome-shaped osteotomy was performed with the use of a reciprocating power saw. After the osteotomy was completed, the distal osseous fragment was displaced medially to improve lateral femoral head coverage. Two 1.8-mm-diameter Kirschner wires were inserted from the proximal part of the ilium into the distal part of the ischium, and the freed greater trochanter was reduced and fixed with two 6.5mm absorbable screws (Figure 3). A hip spica cast was applied for four weeks and the Kirschner wires were removed six weeks postoperatively.

**Figure 4:**
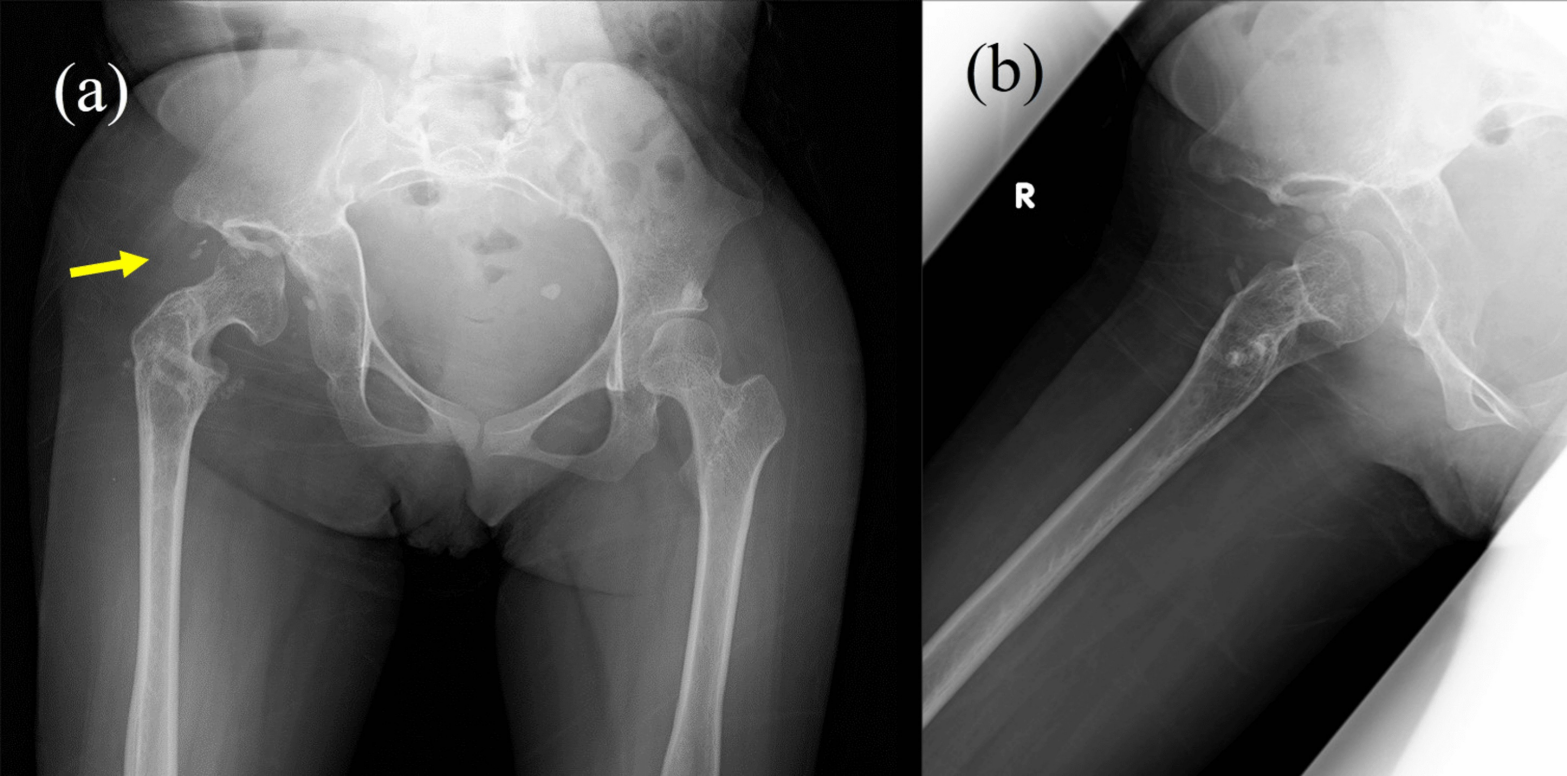
Six years after the surgery, a) AP and b) Lauenstein view reveal no dislocation of the femoral head.

Six years after the operations, the patient had an excellent clinical result. She had no limitation of activities of daily living and sports activity since the hip joint has remained stable without dislocation. No dislocation was detected on an X-ray (Figure 4).

## Discussion

Patients with spina bifida often encounter challenges related to activities of daily living, such as transfers and urination, and may need nursing care. It is crucial to gain a precise understanding of the difficulties the patient faces in their daily life. Surgical intervention for hip joint dislocation in spina bifida patients aims to mitigate dislocation, stabilize the joint, and enhance functionality. Surgery may be recommended when conservative treatments, such as bracing, physical therapy, or medication, prove ineffective in managing hip dislocation. The choice of a specific surgical approach and technique depends on the patient's unique anatomy, the severity of the hip dislocation, and any accompanying medical conditions. It is imperative to discuss the risks and benefits of surgery with both the patient and their family before proceeding.

Some studies have reported improved quality of life in spina bifida patients with lumbar-level spinal paralysis and dislocated hips following femoral varus osteotomy and/or pelvic osteotomy [7]. Wright JG mentioned that individuals with lesions above L4 have a low likelihood of community ambulation beyond adolescence. The literature does not support surgical treatment for hip dislocation in these cases. A majority of the children with spina bifida at L4 level and below can walk within their home or community [8]. Conklin MJ et al. do not advocate routine hip surveillance or surgical intervention for hip subluxation/dislocation, though patients with a low lumbar or sacral lesion and unilateral dislocation may be considered exceptions and should be assessed individually [9,10]. However, there is a dearth of reported cases of recurrent hip dislocation associated with a dysplastic acetabulum in spina bifida patients with complete paralysis at the thoracic level, and there is no consensus regarding the optimal treatment for this condition. In this case study, appropriate surgical treatment and osseous support may have demonstrated the potential to prevent hip dislocation even in patients with complete paralysis. It is crucial to offer comprehensive explanations and ensure that the patient and their family appreciate the controversial nature of treating paralyzed hips.

To the best of our knowledge, this report is the first to document the successful treatment of a patient with thoracic-level spina bifida with complete paralysis of the lower extremities. A pelvic osteotomy was performed for a re-dislocation after a femoral osteotomy and a good outcome was achieved. The patient and their family expressed satisfaction with the surgical outcome. Surgical intervention could be recommended for recurrent hip dislocation for lower limb paralysis due to any degree of spina bifida. However, given that this study reports only a single case, the results should be interpreted with caution. Before opting for surgical treatment, a thorough assessment of the patient's goals and living environment should be conducted. Further research is imperative to ascertain the effectiveness of surgical treatment for this uncommon disorder.

## Conclusions

There is no consensus regarding the treatment for recurrent hip dislocation in a patient with complete lower extremity paralysis due to spina bifida at the thoracic level. To the best of our knowledge, this is the first report of successful treatment of recurrent hip dislocation in a patient with complete lower extremity paralysis due to spina bifida at the thoracic level, performing a combination of femoral varus osteotomy and pelvic osteotomy. In this case study, appropriate surgical treatment with osseous support of a femoral head demonstrated the potential to prevent hip dislocation even in patients with complete lower paralysis. Prior to surgical intervention, a comprehensive assessment of the patient's physical condition, patient's goals, and living environment is essential. Further research is imperative to ascertain the effectiveness of surgical treatment for this uncommon disorder.
